# Seeded Fibrillation as Molecular Basis of the Species Barrier in Human Prion Diseases

**DOI:** 10.1371/journal.pone.0072623

**Published:** 2013-08-20

**Authors:** Lars Luers, Oliver Bannach, Jan Stöhr, Michael Marius Wördehoff, Martin Wolff, Luitgard Nagel-Steger, Detlev Riesner, Dieter Willbold, Eva Birkmann

**Affiliations:** 1 Institute of Physical Biology, Heinrich-Heine University, Düsseldorf, Germany; 2 Institute of Complex Systems (ICS-6), Research Centre Juelich, Juelich, Germany; 3 Institute for Neurodegenerative Diseases, University of California San Francisco, San Francisco, California, United States of America; Colorado State University, College of Veterinary Medicine and Biomedical Sciences, United States of America

## Abstract

Prion diseases are transmissible spongiform encephalopathies in humans and animals, including scrapie in sheep, bovine spongiform encephalopathy (BSE) in cattle, chronic wasting disease (CWD) in deer, and Creutzfeldt-Jakob disease (CJD) in humans. The hallmark of prion diseases is the conversion of the host-encoded prion protein (PrP^C^) to its pathological isoform PrP^Sc^, which is accompanied by PrP fibrillation. Transmission is not restricted within one species, but can also occur between species. In some cases a species barrier can be observed that results in limited or unsuccessful transmission. The mechanism behind interspecies transmissibility or species barriers is not completely understood. To analyse this process at a molecular level, we previously established an *in vitro* fibrillation assay, in which recombinant PrP (recPrP) as substrate can be specifically seeded by PrP^Sc^ as seed. Seeding with purified components, with no additional cellular components, is a direct consequence of the “prion-protein-only” hypothesis. We therefore hypothesise, that the species barrier is based on the interaction of PrP^C^ and PrP^Sc^. Whereas in our earlier studies, the interspecies transmission in animal systems was analysed, the focus of this study lies on the transmission from animals to humans. We therefore combined seeds from species cattle, sheep and deer (BSE, scrapie, CWD) with human recPrP. Homologous seeding served as a control. Our results are consistent with epidemiology, other *in vitro* aggregation studies, and bioassays investigating the transmission between humans, cattle, sheep, and deer. In contrast to CJD and BSE seeds, which show a seeding activity we can demonstrate a species barrier for seeds from scrapie and CWD *in vitro*. We could show that the seeding activity and therewith the molecular interaction of PrP as substrate and PrP^Sc^ as seed is sufficient to explain the phenomenon of species barriers. Therefore our data supports the hypothesis that CWD is not transmissible to humans.

## Introduction

Prion diseases are transmissible spongiform encephalopathies (TSE), which occur in mammals, including scrapie in sheep, bovine spongiform encephalopathy (BSE) in cattle, chronic wasting disease (CWD) in deer and Creutzfeldt-Jakob disease (CJD) in humans. The infectious agent, referred to as prion, is composed mainly, if not exclusively, of the pathological isoform of the host-encoded prion protein (PrP). The key molecular event in prion diseases is the misfolding and aggregation of the cellular isoform of PrP, denoted as PrP^C^, into the pathological isoform, PrP^Sc^ [1]. This conversion is accompanied by a shift from α-helical dominated to β-sheet-rich secondary structure which results in insolubility and aggregation. The aetiology of prion diseases can be spontaneous, genetic or infectious. In analogy, CJD can occur spontaneously (sCJD), as genetic form (fCJD) or as variant form (vCJD), which was transmitted by BSE-afflicted cattle in the 1980s [2].

Scrapie was first described several hundred years ago and transmissibility among sheep could be shown in the 1930s [3]. Since the BSE epidemic it has become apparent that prions can be transmitted from one species to another e.g. from sheep to cattle [4]. The discovery of interspecies transmission became even more alarming with the incidence of vCJD, which has been shown to be caused by the consumption of BSE contaminated food [5].

Interestingly, there are transmission barriers between some species leading to no or at least a much less effective transmission. Scrapie, for instance, is known for almost three centuries and yet no case of transmission from sheep to humans has been described. In addition, experimental transmission studies in transgenic rodent models have been found to result in prolonged incubation time and an incomplete attack rate – a phenomenon which is referred to as the species barrier [6]. A recent study reported, that the species barrier has a tissue specificity, adding even more complexity [7]. Beyond this, it is still uncertain if the species barrier is a result of an ineffective route of infection, cellular cofactors, incompatibility between pathogenic PrP^Sc^ and the host-encoded PrP^C^ or a combination thereof.

Several mechanisms have been proposed to describe the conversion of PrP^C^ to PrP^Sc^ [8]. One widely accepted is the seeded polymerization model [9]–[12]. To analyse this conversion process at a molecular level, we have previously developed an *in vitro* assay which is able to generate PrP fibrils *de novo* [13], [14]. Recombinantly expressed PrP (recPrP) is suspended in physiological sodium phosphate buffer and very low concentrations of sodium dodecyl sulfate (SDS), establishing a well-defined pre-amyloid state [11], [15]. Based on this, we also established a seeded fibrillation assay, which is suitable to analyse PrP fibrillation mechanistically, as it is only consisting of recPrP and prion seeds, derived from brain tissue precipitated by phosphotungstic acid (NaPTA) [16], [17]. No additional cellular extracts are needed.

In an earlier study, Caughey and colleagues studied the conversion of cellular PrP from N2a cells by infectious brain samples, applying close to stoichiometric ratios of PrP^Sc^ and PrP^C^ [18]. For heterologous systems a “relatively ineffective conversion of human PrP^C^ by PrP^BSE^” was shown. Further, “CWD transmissions to humans would be as limited by PrP incompatibility as transmission of BSE or scrapie to humans” [19].

In previous studies, we combined seeds and recPrP from different species. We could show, that our assay perfectly matches the known *in vivo* transmissibilities between sheep, cattle, mice and hamster [20], [21]. With our seeded fibrillation assay we could model the interspecies transmission from sheep to cattle and from sheep to Syrian hamster (SHa), while the species barrier between sheep and mouse was confirmed.

In a similar seeding approach, but with different partially denaturing incubation conditions, “promiscuous seeding” between hamster seeds and mouse PrP^C^ was observed [22].

In the present study, we extended the seeded fibrillation assay to the cervid and human system to address public health concerns of transmission of CWD prions to human by consumption of contaminated food.

## Materials and Methods

### Cloning and expression

Genes coding the human PrP 90-231 (UniProtKB/Swiss-Prot: P04156) and cervid recPrP 89-231 (UniProtKB/Swiss-Prot: Q7JIQ1) were cloned from synthetic vectors (pMK; Mr. Gene) into expression vector pET 16b and then electro transformed into *E. coli* BL 21 Rosetta (DE3) pLysS cells (Merck/Novagen). Purified plasmids of selected clones were sequenced (StarSEQ) for sequence verification. An *E.coli* stock solution of each clone was stored at –80°C. After sequencing, only stocks with verified sequence have been used for further experiments.

Human recPrP 90–231 (huPrP) and cervid recPrP 89–231 (cerPrP) expression was induced by adding 1 mM Isopropyl-β-D-thiogalactopyranosid (IPTG; Fermentas) to a 1 L *E.coli* culture with a cell density of OD_600_ = 1.5. PrP expression was performed at 30 °C and 200 rpm for 20 h. Cells were harvested by centrifugation at 4,000 rcf (Beckman Coulter Allegra X15).

### Purification

1 g *E.coli* cell pellet was suspended in 5 ml phosphate buffered saline (PBS; 0.01 M phosphate; 0.138M NaCl; 0.027 M KCl, [pH 7.4]),containing protease inhibitor (complete mini EDTA free; Roche), 10 µg/ml DNAse I grade II and 20 mM MgCl_2_ (Sigma-Aldrich) at RT. Cells were lysed by application of a French press (Constant Systems; One Shot Cell Disruptor) at 2.5 kbar, the resulting lysate was pelleted. Pellets were suspended in 5 ml 8 M guanidinium chloride (GndHCl; Sigma-Aldrich), 12.5 mM TrisHCl [pH 8] (Sigma-Aldrich) and incubated over night at 4°C in an head-over-tail rotator. The resulting suspension was centrifuged at 50,000 rcf and 10°C. The supernatant was used for metal ion affinity chromatography (IMAC).

IMAC was performed on an Äkta prime system (GE Healthcare (Amersham bioscience) using 1 ml NiNTA columns (NM; Protino). Equilibration/wash buffer was 6 M GndHCl, 0.1 M NaP_i_ (NaH2PO4/Na2HPO4; [pH 7.4]); 0.1 M TrisHCl 0.15 M NaCl and 0.02 M Imidazole [pH 8]. Elution buffer equalled equilibration buffer with NaCl and imidazole concentration raised to 0.5 M.

Elution peak was incubated with 10 mM dithiothreitol (DTT; Sigma-Aldrich) for 1 hour at RT. A buffer exchange was performed (6 M GndHCl; 12.5 mM TrisHCl; [pH 8]) in centricon filtration units (Centricon, Millipore, 10 kDa MWCO). The protein solution was then incubated for 24 hours at RT with 10 mM oxidised glutathione (GSSG; Sigma-Aldrich) and 1 mM reduced glutathione (GSH; Sigma-Aldrich). Buffer was exchanged again to 6 M GndHCl and the protein was refolded by rapid dilution with 10∶1 1.1x Factor Xa cleavage buffer. At this point, protein concentration was roughly estimated by absorption spectrometry using calculated molar extinction coefficients. His tag was removed by digestion with 0.1 U factor Xa (Merck/Novagen) per µg PrP. After Cleavage at RT for 24 hours, final purification by reversed phase high performance liquid chromatography (rp-HPLC) followed.

Rp-HPLC was performed on a HPLC system (Knauer WellChrom K-1001) with 50 ml Eurosil 300 C4 column. Running a gradient of acetonitrile and ultrapure water at a flow rate of 5 ml/min the recPrP was eluted at 40–42% acetonitrile. Eluted protein was then lyophilised and dissolved in stock buffer containing 10 mM NaP_i_ [pH 7.4] and 0.1% SDS. After estimation of protein concentration by BCA-assay (Thermo Scientific; Pierce) and absorption spectrometry protein stocks were stored at –80°C.

### Circular Dichroism (CD) spectroscopy

CD measurements were carried out in a Jasco J-815 CD spectropolarimeter at a fixed temperature of 20°C. Protein samples were diluted to a final concentration of 18 µM. Prior to CD measurements, 250 mM NaCl was added and the solution was adjusted to SDS concentrations between 0.01% and 0.2% SDS. CD spectra were recorded between 260 and 190 nm using a step size of 0.5 nm and 10 accumulations. Buffer spectra have been recorded with similar settings and subtracted afterwards.

### Analytical ultracentrifugation

Ultracentrifugation was performed in an Optima XL-A analytical ultracentrifuge (Beckman Coulter) with absorbance optics using an An-50 Ti rotor with aluminium 2-channel centerpiece cells. HuPrP at 2.5 and 5 µM concentration was analysed after incubation for 5 days at 37°C and 650 rpm in fibrillation buffer (10 mM NaP_i_ [pH 7.4]; 250 mM NaCl; 0.02% SDS) to reach pre-amyloid state. Sedimentation velocity centrifugation was conducted at 40,000 rpm (128,794 rcf at 7.2 cm) and 20°C. Scans were recorded in intensity mode at 230 nm at 6 min intervals with a radial resolution of 0.003 cm and transformed to absorbance scans afterwards. For data evaluation a non-interacting independent species model from the software package Ultrascan 3 (www.ultrascan.uthscsa.edu) was applied using a vbar of 0.7102 cm^3^/g based on the sequence of huPrP [23]. The fitting procedure consisted of a 2-dimensional spectrum analysis followed by a genetic algorithm and was finalised by Monte Carlo statistics [24]–[26]. Reported *s*-values are corrected for water at 20°C. Calculations were performed on the UltraScan LIMS cluster at the Bioinformatics Core Facility at the University of Texas Health Science Center at San Antonio. XSEDE resources were supported by NSF Teragrid Grant #MCB070038 (to Borries Demeler).

### *De novo* SDS-based fibrillation assay

Analysis of kinetics of PrP fibrillation is based on protocols published previously [14]. Samples have been measured in a microtiterplate (MTP) in a fluorescence reader (TECAN; M200Pro) at 37°C. Every 30 minutes, fluorescence emission was measured at 482 nm (λ_ex_: 445 nm) averaging 25 single measurements for every well and time point. Between measurements (max. 5 minutes), the MTP was agitated rigorously by linear shaking at an amplitude of 2 mm (≈600 rpm) for at least 25 minutes. 9 µM PrP in NaPi containing 250 mM NaCl, 0.01–0.05% SDS and 5 µM Thioflavin T (ThT) at a final volume of 300 µl was measured in black 96-U-well microtiterplate (Nunc) sealed with adhesive foil (Thermo Scientific/Nunc, optical grade). Measurements for the *de novo* SDS-based fibrillation assay were conducted for at least three weeks.

### TIRF microscopy of ThT stained huPrP fibrils

Total internal reflection fluorescence (TIRF) microscopy was performed using a Leica AM TIRF MC equipped with a HCX PL APO 100×1.47 oil immersion objective (Leica microsystems). Prior to TIRF analysis, cover slips (25×6×0.17 mm; Menzel Gläser; Thermo Fisher Scientific) were cleaned with 2% Hellmanex III for 15’ in an ultrasonic bath. After washing 10 times with in ultrapure water and once with absolute ethanol for 1’, slips were dried under a stream of nitrogen gas. For immobilization of aggregates on a glass surface 5 µl of *de novo* generated huPrP fibrils (from a monomer solution containing 9µM huPrP) have been dried on a cover slip at RT. 10 µl of ThT solution (10 µM) was added and fibrils were visualised in TIRF mode (penetration depth 150 nm) with λ_ex_: 405 nm. ThT fluorescence was detected by EM-CCD camera (Model 9100-2, Hamamatsu) at an exposure time of 1 s. Images were post-processed by adjusting contrast and brightness in open source imaging software ImageJ (available at http://rsb.info.nih.gov/ij; Wayne Rasband, National Institutes of Health, Bethesda, MD).

### Transmission electron microscopy (TEM) of negative-stained huPrP fibrils

5 µl of fibrillar recPrP (confirmed by ThT fluorescence) was placed on a glow discharged grid and left to adsorb for 1 minute. The sample was washed with 50 µl of 0.1 and 0.01 M ammonium acetate and stained with 50 µl 2% ammonium molybdate. Dried samples were analysed with a Tecnai F20 transmission electron microscope at 80 kV.

### PrP^Sc^ pre-purification by NaPTA precipitation

NaPTA**-**precipitation of PrP^Sc^ from brain tissue is a standard procedure for the preparation of infectious prion samples. The purification protocol is described in [20] and was performed identical for each sample used within this work. 700 µl of 5% brain homogenate (10% in case of sheep) in homogenisation buffer (1× PBS; 4% Sarcosyl; proteinase inhibitor) was thawed for 15 minutes at 37°C and 650 rpm. Brief centrifugation in a table top centrifuge at 2,000 rcf at 20°C. Addition of 500 µl 4% NaPTA [pH 7.4] to 500 µl of the resulting supernatant. 60 minutes incubation at 37°C and 650 rpm followed by 30 minutes of centrifugation at 14,000 rcf at 20°C. Removal of supernatant and addition of 200 µl 2% NaPTA [pH 7.4] and 2% N-lauroylsarcosine (sarcosyl) followed by a brief ultrasonification (50W; 5 seconds). This centrifugation step followed by removal of the supernatant and ultrasonification in exchanged buffer is repeated three times with 200 µl of 100 mM NaPi [pH 7.4] followed by 200 µl and finally 100 µl of 10 mM NaPi [pH 7.4]. The resulting seed was used for the following seeded fibrillation assay.

Corresponding to the respective diseases NaPTA precipitate are denoted as seed: CJD seed; BSE seed; scrapie seed and CWD seed. Seeds from brain tissue of healthy animals are denoted as controls.

Samples of brain tissue were kindly provided by Oliver Andreoletti, INRA ENVT (classical scrapie; Medulla), Martin Groschup, Bundesforschungsanstalt für Viruskrankheiten der Tiere (classical BSE, Medulla), Neil Cashman, Canadian Cooperative Wildlife Health Centre (CWD; White-tailed deer) and Hans Kretzschmar, Institut für Neuropathologie und Prionforschung der LMU (sCJD)

### Seeded SDS-based fibrillation assay

The seeded fibrillation assay is based on a previous protocol [11], [20] and was conducted as described in paragraph “*De novo* SDS-based fibrillation assay” with the following exceptions: (i) SDS concentration was fixed to 0.02% SDS, (ii). Time frame for measurements was maximum seven days and (iii) a 50 µl seed (prepared as described in paragraph “PrP^Sc^ purification by NaPTA precipitation”) was added.

Seeding activity was calculated by dividing the average of relative fluorescence of samples with PrP^Sc^ positive seeds and PrP^Sc^ negative seeds within 80–100 h (plateau phase). Homologous combinations of seed and substrate (e.g. CJD seed in huPrP) always served as positive controls for heterologous seeding experiments.

### Statistical analysis of multiple seeding experiments

To test for statistical significant differences between seeding experiments of human seeds in huPrP (intraspecies case) and ovine, bovine or cervid seeds in huPrP (interspecies case) we reproduced these experiments several times and calculated the seeding activity for those combinations. Data is illustrated as Tukey’s box plot. An ANOVA with additional Tukey’s range test was performed with the log_2_ of each value (seeding activity of each individual experiment), which is necessary to get normally distributed values when analysing fluorescence data near zero.

### Ethics Statement

The study was submitted to the ethics committee of the medical faculty of the Heinrich-Heine-University of Düsseldorf (Ethikkommission an der Medizinischen Fakultät der Heinrich-Heine-Universität Düsseldorf). The Ethics committee waived approval, since there were no ethical concerns about this study. Human brain samples were obtained from the Society for prion research Germany (Gesellschaft zur Förderung der Prionforschung e.V. c/o Zentrum für Neuropathologie & Prionforschung, München). Patients’ next to kin gave their written consent for autopsy. All data were analysed anonymously. Bovine brain samples were obtained from the national reference laboratory for BSE in Germany (Bundesforschungsanstalt für Viruskrankheiten der Tiere) Ovine brain samples were obtained from the National Veterinary School of Toulouse (École Nationale Vétérinaire de Toulouse). Cervid brain samples were obtained from the Canadian Cooperative Wildlife Health Centre (Western College of Veterinary Medicine; University of Saskatchewan).

## Results

In order to analyse the molecular mechanism of fibrillation of human recombinant PrP (huPrP) in our SDS based fibrillation assay, we determined the particular concentrations of recPrP and SDS, which trigger *de novo* fibrillation of PrP after several weeks. In contrast, fibrillation in the presence of seeds can be observed within days or even hours, underlining the efficiency of seeding. The conformation of PrP under conditions which enable fibrillation is called pre-amyloid state [11]. The pre-amyloid state of huPrP was characterised by biophysical methods including circular dichroism (CD) spectroscopy and analytical ultracentrifugation. *De novo* and seeded fibril formation was monitored by ThT fluorescence and fibrils generated were visualised by ThT-stained TIRF and electron microscopy. To analyse the species barrier phenomenon a seeded fibrillation assay on the basis of huPrP was established. NaPTA precipitates of brain tissue of different prion infected species as seeds were combined with human recPrP as substrate.

### Pre-amyloid state of huPrP

**Figure 1:**
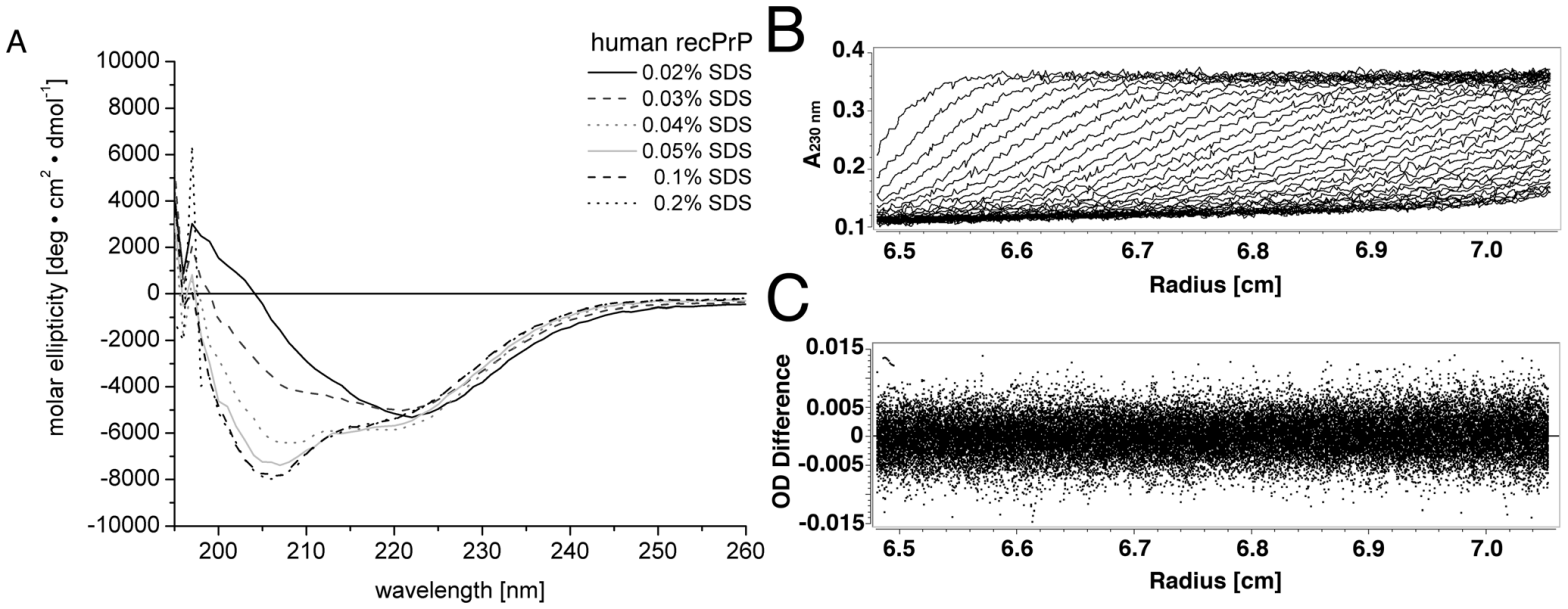
Pre-amyloid state of huPrP – CD spectroscopy and sedimentation velocity analysis of huPrP. CD spectra (**A**) of huPrP with varying concentrations of SDS (0.02–0.2%) have been recorded directly after adapting the SDS buffer conditions. CD spectra show a shift from α-random dominated towards β-sheet rich secondary structure with decreasing concentration of SDS. (**B and C**) A sedimentation velocity experiment at 20°C and 40.000 rpm was performed to determine the state of oligomerisation of huPrP. Fig. 1 **B** shows the successive radial absorbance scans of 5 µM huPrP, which have been corrected for time and radially invariant noise. For clarity only every 3^rd^ scan was plotted. Fig. 1 **C** shows the deviation of measured data points from the fitted sedimentation boundary after the fitting procedure using Ultrascan 3. The absorption difference at 230 nm is plotted in relation to the distance from the rotor axis. The RMSD of the fit was 0.00322 A_230_.

CD spectroscopy has been applied to determine the secondary structure of the pre-amyloid state of human recPrP 90-231 (huPrP). A concentration series of SDS ranging from 0.01–0.2% was prepared containing 18 µM huPrP. In SDS concentrations ranging from 0.04–0.2% a mixture of α-helical and random-coil secondary structure was observed, as indicated by a pronounced minimum at 208 nm. In SDS concentrations ranging from 0.02–0.03%, the spectrum shifts towards a minimum at 220 nm with a zero-crossing at higher wavelength, suggesting a β-sheet dominated secondary structure (Fig. 1 A).

Analytical ultracentrifugation in sedimentation velocity mode was used to analyse the state of oligomerisation of the pre-amyloid state of huPrP. Data analysis of the 5 µM huPrP sample revealed two species. The most prominent species (∼65%) has an *s*-value of 3.15±0.01 S and a frictional ratio of 1.5. The quality of the fit was confirmed by the low root mean square deviation (RMSD) value of 0.00322 A_230_ (Fig. 1 B and C).

This species with a calculated molecular weight of 40.7±0.25 kDa is interpreted as a huPrP dimer. The huPrP dimer has a calculated molecular weight of 32.6 kDa. Difference in calculated molecular weight from sedimentation velocity analysis is interpreted as the solvation of the protein and the binding of SDS molecules to the protein complex.

A second species has an *s*-value of 0.186±0.003 S and a frictional ratio of 1. The corresponding molecular weight is far too low to be interpreted as monomeric PrP.

It can be rather interpreted as an artefact arising from a non-zero baseline, which might be caused most probably by SDS.

Lowering the protein concentration to 2.5 µM huPrP did produce very similar results. A small species corresponding to the non-zero baseline and one species at 3.0±0.02 S with 40.0±0.4 kDa based on a frictional ratio of 1.5 corresponds to the dimeric huPrP (RMSD of 0.0034 A_230_). Again no species corresponding to monomeric huPrP was detected.

### *De novo* fibrillation of huPrP

To analyse *de novo* fibril formation of huPrP, samples containing 3 and 9 µM huPrP were incubated with 0.02–0.05% SDS for three weeks at 37°C with constant agitation. Fibril formation was monitored by Thioflavin T (ThT) fluorescence, which serves as an specific indicator for the presence of amyloid fibrils.

**Figure 2:**
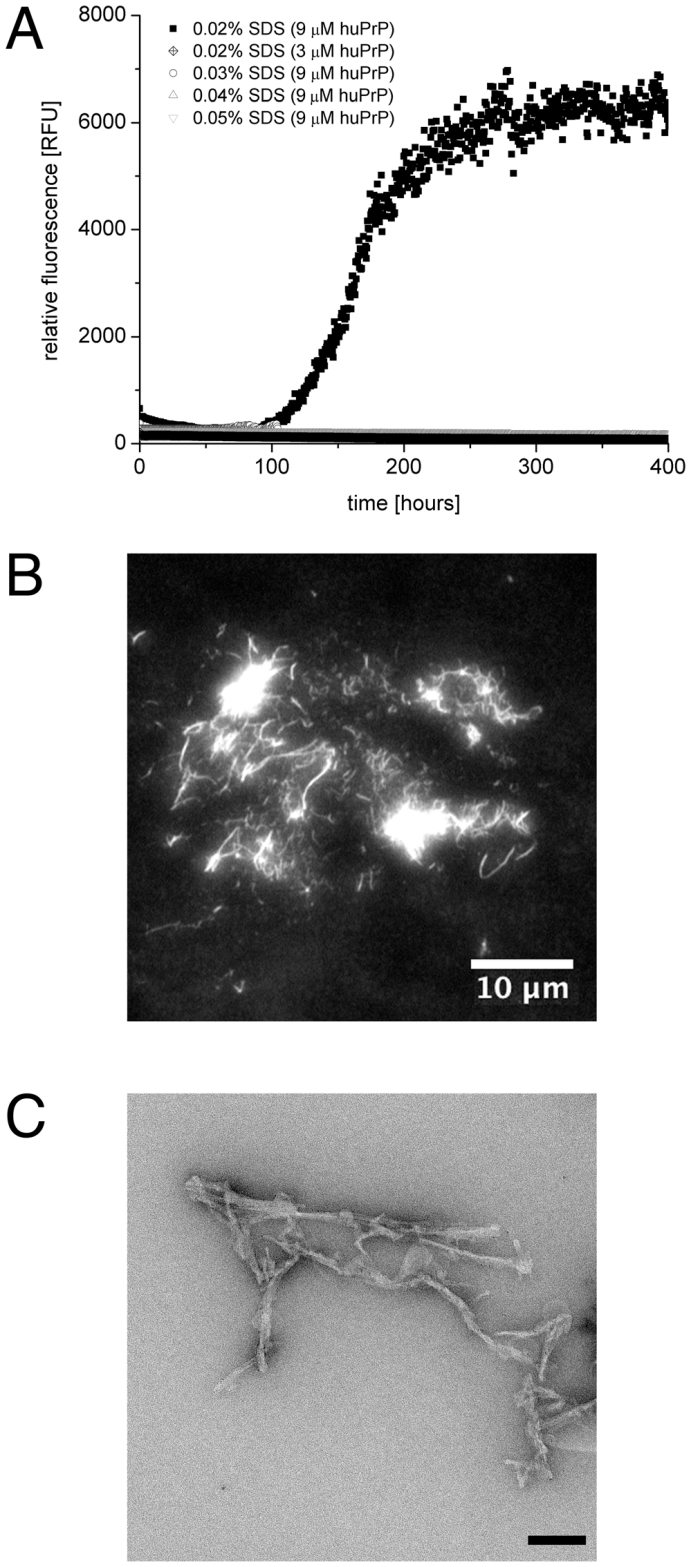
*De novo* fibrillation of huPrP – ThT kinetics and TIRF micrographs. *De novo* fibrillation is shown in (**A**), an amyloid specific increase in ThT fluorescence is only observable at 0.02% SDS and 9 µM huPrP. (**B**) *De novo* generated amyloid fibrils (plateau phase) have been stained by ThT and visualised by TIRF microscopy. (**C**) Ultrastructure of negative-stained *de novo* huPrP fibrils obtained by transmission electron microscopy. Bar equals 100 nm.

Fibril formation depends both on SDS and huPrP concentration. Samples containing 9 µM huPrP and 0.02% SDS showed an increase in ThT fluorescence within 400 hours (Fig. 2 A). Within this time frame, all other combinations did not show increased ThT fluorescence. The kinetics follow a sigmoid curve with a lag phase of about 100 hours, reaching a plateau at about 250 hours of incubation.

Morphology and size of huPrP aggregates were analysed by TIRF and electron microscopy after ThT staining. ThT stained huPrP aggregates with an estimated average length of about 1 to 5 µm were found. Aggregates of fibrillar character and also bigger aggregates of highly ThT-stained protein are the most abundant species visible (Fig. 2 B). Similar results were obtained by negative-stain TEM of *de novo* huPrP fibrils. HuPrP aggregated into prototypical amyloid fibrils with an estimated diameter between 15 to 20 nm (Fig. 2 C).

### Seeded fibrillation of huPrP

**Figure 3:**
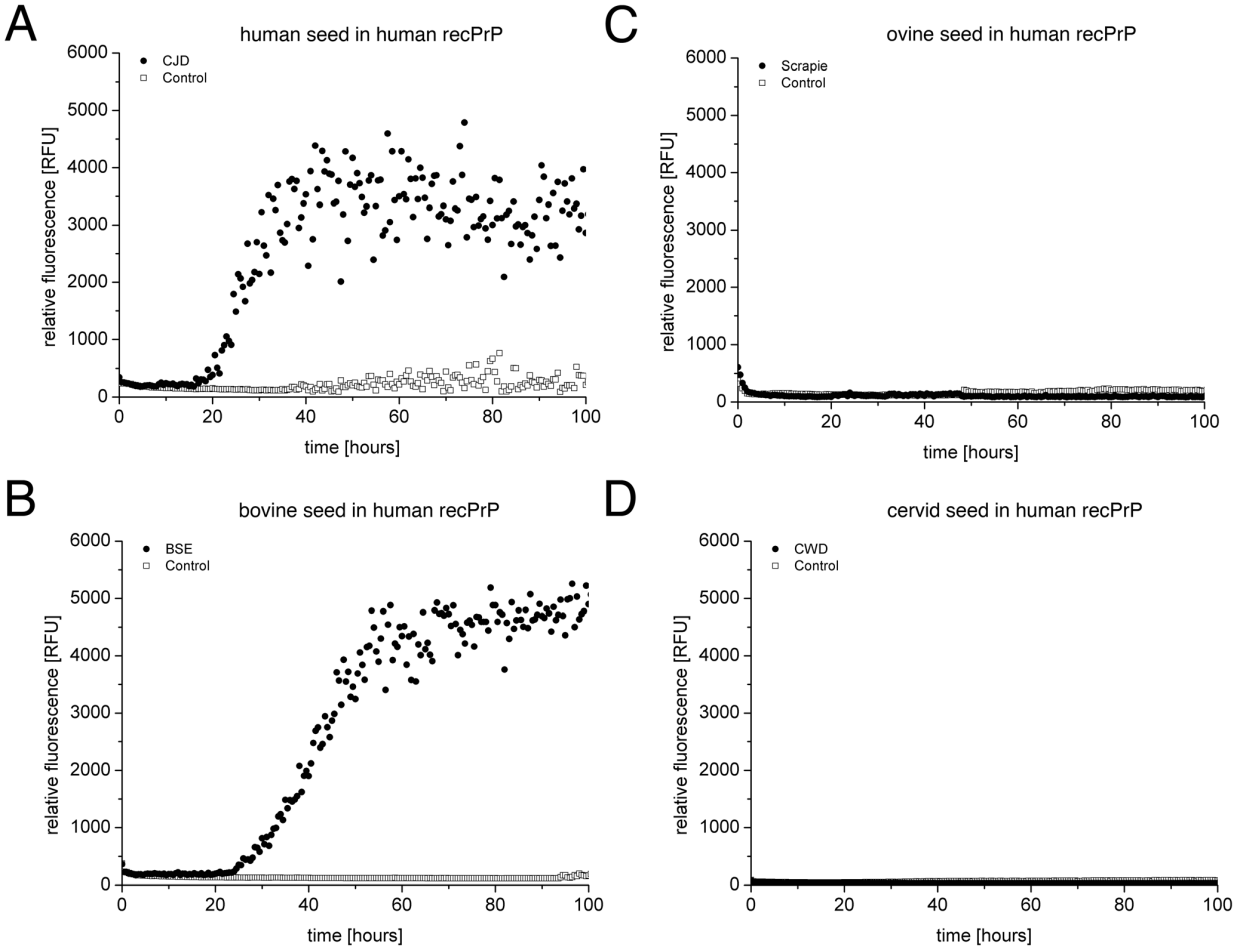
Seeded fibrillation of huPrP. Different combinations of seeds from NaPTA precipitated brain homogenates of either diseased or control animals and 3 µM huPrP as substrate within ThT amyloid seeding assay. Time dependent ThT fluorescence is shown for 3 µM huPrP in combination with seeds from CJD-affected human brain (**A**), scrapie-affected sheep brain (**B**), BSE-affected bovine brain (**C**), and CWD-affected deer brain (**D**) (closed circles). The controls (open rectangles) were prepared from the respective non-diseased brain material from human (A) and animals (**B; C and D**).

CJD seeds and huPrP as substrate were analysed as an intraspecies combination which serves as a positive control. While brain samples of confirmed CJD victims are able to seed fibrillation, age-matched controls of non-CJD samples did not seed fibrillation (Fig. 3 A). The kinetics show an increase in ThT-specific fluorescence after a lag phase of about 20 hours which is about five times shorter than in the *de novo* fibrillation assay (Fig. 2 A). A concentration of 0.02% SDS was used in the seeding assay to promote the formation of the pre-amyloid state. However, to suppress *de novo* fibril formation, only 3 µM huPrP was used in the seeded fibrillation assay.

### *De novo* and seeded fibrillation of cerPrP

**Figure 4:**
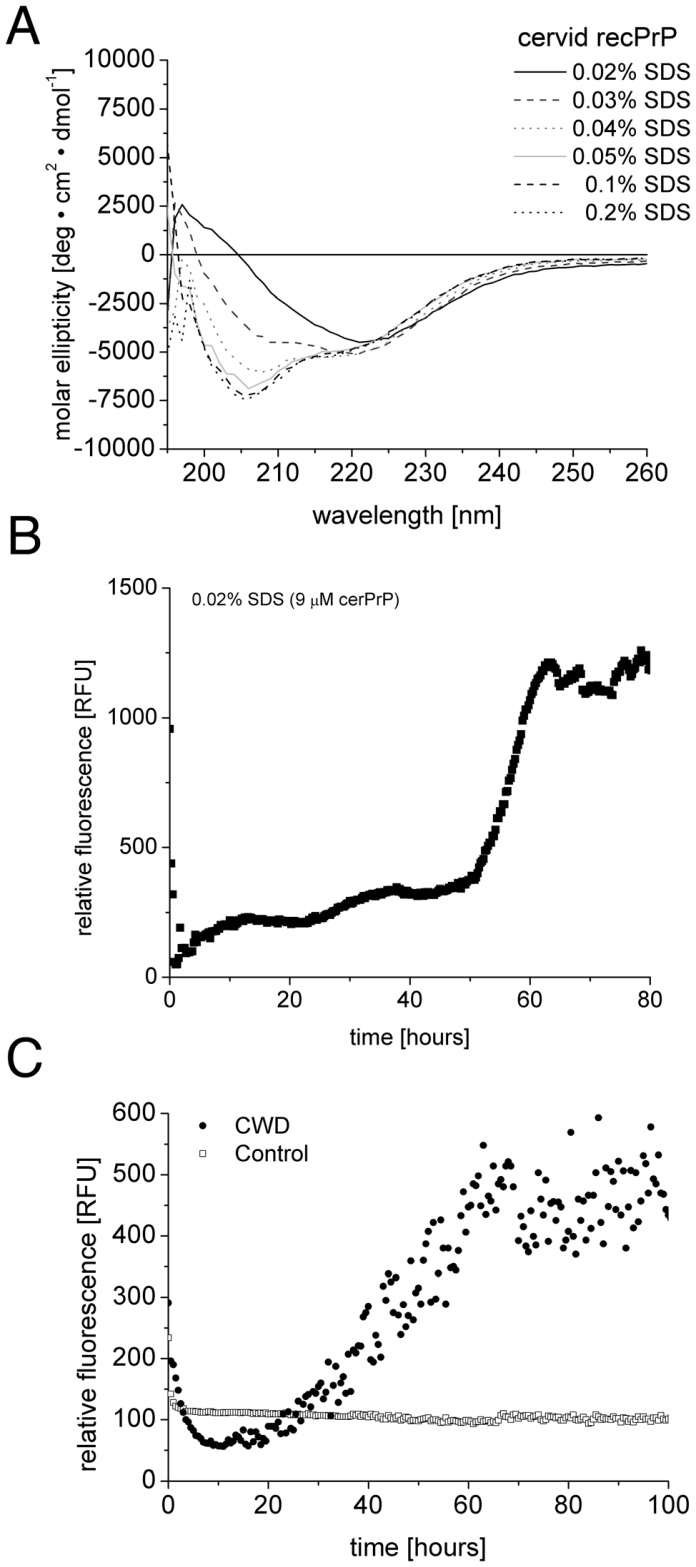
Pre-amyloid and amyloid state of cerPrP. CD spectra (**A**) of cerPrP with different concentrations of SDS (0.02–0.2%) have been recorded directly after adapting the SDS buffer conditions. CD spectra show a shift from α-random dominated towards β-sheet rich secondary structure with decreasing concentration of SDS. (**B**) *De novo* fibrillation of cerPrP, an amyloid specific increase in ThT fluorescence is observable at 0.02% SDS and 9 µM cerPrP. (**C**) cerPrP (3 µM) in combination with seeds from CWD brain or control brain.

To analyse a species barrier between cervids and humans, fibril formation of cerPrP was established in the *de novo* fibrillation assay as described above. As shown for huPrP, a similar shift in secondary structure within the SDS concentration series was also found for cerPrP (Fig. 4 A). Also *de novo* fibril formation could be shown for 9 µM cerPrP in 0.02% SDS (Fig. 4 B), the corresponding CD spectra indicate a β-sheet rich secondary structure. The seeding ability of CWD seeds and controls were analysed in 3 µM cerPrP in 0.02% SDS. An increase of ThT fluorescence within 40 to 60 hours was detected only in the sample with CWD seeds (Fig. 4 C).

### Fibrillation of huPrP seeded by animal seeds

We have established the huPrP fibrillation assay in order to test whether fibrillation of huPrP can be seeded by scrapie, BSE or CWD seeds. The combination of seed and substrate of one and the same species always served as positive control. Seed prepared out of non-infected brain tissue always served as negative control. The corresponding studies using BSE and scrapie seeds in combination with recPrP from cattle and sheep, respectively, were described earlier [20], [21]. In these studies, it could be shown that seeds from BSE and scrapie are able to promote fibril formation of bovine or ovine recPrP, respectively. Scrapie seeds were also able to seed the fibril formation of bovPrP, representing a heterologous combination of seed an substrate. On the contrary, species barriers could be shown for hamster seed in recombinant mouse PrP and BSE seed in recombinant hamster PrP, i.e. no interspecies seeding activity could be shown with these combinations.

In this study, BSE seeds and control seeds were combined with huPrP as substrate, showing an increase in ThT fluorescence only in samples containing BSE seed, whereas no increase was observed for control seeds (Fig. 3 B). This seeding activity, shown in our *in vitro* assay, matches the experimentally shown interspecies transmission *in vivo* [5].

However, no seeding activity was observed, when scrapie prions were used with huPrP as substrate. No increase in ThT fluorescence was detectable for NaPTA preparations of scrapie-positive or scrapie-negative tissue (Fig. 3 C).

Finally, we explored the question if CWD seeds are able to seed fibril formation of huPrP. As shown above, huPrP can be converted with CJD seeds (Fig. 3 A) and cerPrP with CWD seeds (Fig. 4 C), respectively. In the heterologous combination no increase in ThT fluorescence could be observed, neither in the sample with CWD-seed, nor in the negative controls (Fig. 3 D). Thus, CWD seeds are not able to promote huPrP fibrillation in our *in vitro* assay. This result suggests, that the species barrier between humans and cervids observed *in vivo* (see discussion) results from the direct interaction of cervid prions and human PrP^C^.

**Figure 5:**
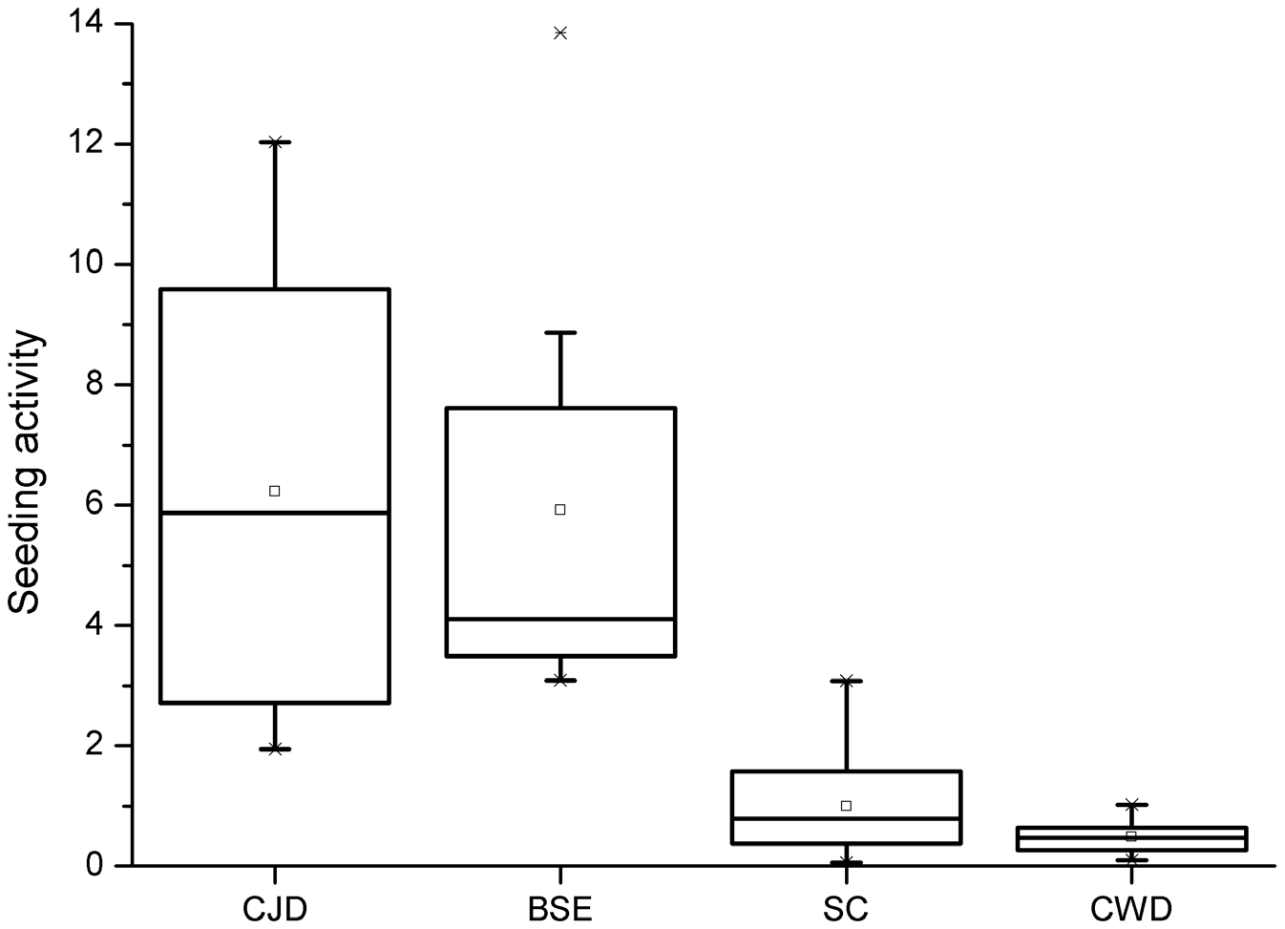
Seeding activity of PrP^Sc^ seeds of different species in huPrP. The box plot shows seeding activity of seeds from the species human, cattle, sheep and deer in huPrP obtained from identical replicates of single seeding experiments. Seeding activity was calculated by dividing the average of relative fluorescence of samples with PrP^Sc^ positive seeds and PrP^Sc^ negative seeds within 80-100 h (plateau phase). For seeds from CJD (N = 7) or BSE (N = 8) material a high seeding activity is observable. For seeds from scrapie (N = 10) or CWD (N = 8) a strong species barrier is apparent.

The quantification of seeding activity (for calculation see materials and methods) of all combinations analysed, is summarised as Tukey’s box plot (Fig. 5). Seeding activity in huPrP was shown for CJD (N = 7) and BSE (N = 8) seeds, whereas no seeding activity could be observed for scrapie (N = 10) or CWD (N = 8) seeds.

To show if these results are significant, we analysed every combination of seed in huPrP at least 7 times. Statistical significance, tested by an ANOVA with Tukey’s Test, is given for the comparison of data sets for species combination human/sheep (p = 0.00026) as well as human/cervid (p = 0.00108).

## Discussion

The molecular dissection of the conversion pathway from PrP^C^ to PrP^Sc^ is still enigmatic and a major focus in prion research. In regard to the species barrier phenomenon the molecular mechanism of the conversion is of particular interest, since the barrier between some species is insurmountable whereas transmission between others is rather effective. In several *in vitro* and *in vivo* studies it was attempted to predict the species barrier between two species by quantification of sequence variations [27]. In recent years, the conformational selection model proposes that the adaptation of two structures is more important than the two sequences [6]. It is assumed that the species barrier is determined by the degree of overlap of permissible structures of PrP^Sc^ between host and donor species. So far, a prognosis of a particular species barrier on the basis of sequence or structural variations could not be achieved.

Several *in vitro* systems, which mimic characteristics of PrP conversion, have been established to induce structural characteristics reminiscent of PrP^Sc^ or to generate and propagate infectivity [28]–[30], rather than to mimic the species barrier.

As mentioned above, intra- and interspecies conversion was assayed by Caughey and colleagues, who converted cellular PrP from N2a cells by infectious brain samples, applying close to stoichiometric ratios of PrP^Sc^ and PrP^C^ [18]. For homologous combinations, a conversion efficiency of up to 25% was demonstrated. For heterologous combination a “relatively ineffective conversion of human PrP^C^ by PrP^BSE^” was shown. Furthermore, “CWD transmissions to humans would be as limited by PrP incompatibility as transmission of BSE or scrapie to humans” [19]. The results of this earlier conversion system led to claims on species barriers in terms of relative effectiveness.

Protein misfolding cyclic amplification (PMCA;[31]), was developed originally as diagnostic tool. Prion seeds are incubated in cellular lysate from non-infected brain tissue, containing a plethora of cellular components. Proteinase K resistant PrP aggregates and, in recent studies, even infectious PrP^Sc^ was generated [32]. Even though strain characteristics of prions were retained in these experiments, it was shown, that PMCA is capable of overcoming naturally existing species barriers [33], [34]. Therefore, seeding activity in PMCA experiments might be too high, due to the order of magnitudes in amplification, to be specific enough for the analysis of the species barrier phenomenon.

Somewhat related to our approach is the QuiC method, in which recombinant hamster PrP (90-231) is used as substrate in a seeding assay without cellular components. Not only prions from hamster, but also from sheep, human and mouse [35]–[37] could be detected applying hamster substrate. Although the QuiC method is very sensitive and applicable for diagnostic purposes [38], a mechanistic characterisation of the species barrier has not been shown, since the overcoming of naturally occurring species barriers seems to be readily done.

In the current and our previous studies, we developed and applied a SDS based *in vitro* assay, in which natural, pre-purified PrP^Sc^ seeds and recPrP substrate are combined to form PrP fibrils with high efficiency in absence of any additional cellular components. Fibril formation could be quantitatively analysed in terms of a direct molecular interaction of seed and substrate, which is a direct consequence of the “prion-protein-only” hypothesis. The dependence of the lag-phase of fibril formation on the monomer, as well as the seed concentration, is in congruence with the mechanism of direct molecular interaction [11], [12]. After the characterisation of the homologous system, prion seeds from different species were combined with recPrP from hamster, mouse, sheep, cattle, deer and human as substrate. In a preceding study, we could show, that our *in vitro* seeding assay is in complete agreement with naturally occurring transmission, as well as species barriers between the species hamster, mouse, sheep and cattle. It was also shown, that the application of the truncated form of recPrP as substrate has no effect on the specificity of the seeding assay [20]. In the present work we have extended the studies to the human and cervid species.

Transmission of CJD within humans has first been described by cases of iatrogenic CJD (iCJD) due to *dura mater* transplants, application of contaminated growth hormone or contaminated medical instruments [39]. The transmission of variant CJD (vCJD) via blood transfusion has been reported in recent years [40], [41]. In our studies CJD seeds and huPrP as substrate were used as an example of intraspecies transmission and served as positive control. As expected, we could show that CJD seeds are able to facilitate fibrillation of huPrP.

Regarding the transmission of prions from other species to humans, epidemiological data could show, that the occurrence of vCJD during the BSE crisis in the late 1990s correlates with emergence of BSE [42]. Several studies, which are based on comparison of lesion profiles, molecular similarities and incubation times in laboratory rodents of the two agents, show a clear connection between BSE and vCJD [43]–[46]. According to these *in vivo* and *in vitro* studies, we showed fibrillation of huPrP induced by BSE seeds. In contrast to claims of “relatively ineffective conversion” mentioned above, we show a significant seeding activity.

A case of a species barrier is described for the transmission from sheep scrapie to humans. Analysis of epidemiological data did not reveal any connection between the incidence of scrapie and CJD [47]–[49]. Additionally, *in vitro* studies and studies with transgenic mice expressing huPrP^C^ could not show transmissibility of scrapie to humans [18], [50]. Regarding this generally accepted species barrier, scrapie prions are not able to seed fibrillation of huPrP.

Since CWD was first described in 1981 in wildlife deer, extensive surveillance studies were started in 1990 [51]. Today, CWD is found in captive and free-ranging cervids in a number of states and provinces throughout North America. The potential transmissibility of CWD to humans is of utmost importance for public health and is subject of current research. Some PMCA studies showed that it is possible to seed human PrP with CWD prions [52]. Also, non-human primates could be infected with CWD prions [53]. In contrast, other *in vitro* and *in vivo* studies indicate the presence of a species barrier between cervids and humans [43], [54]. In one of these studies, the conversion of human PrP^C^ in brain homogenate could only be shown, when CWD seeds were pre-treated under acidic conditions, as stated “possibly by overcoming conformational barriers in partial denaturation of substrate”. In neutral buffer conditions no conversion of human PrP^C^ could be shown. Also bioassays in transgenic mice strengthen the assumption, that CWD is not transmissible to humans [50], [55]–[57]. Moreover, epidemiological studies cannot find a correlation between CWD and human prion diseases [43], [58]. Our studies do not show any seeding activity of CWD seeds in huPrP. This corresponds to most of the studies mentioned above, which shows that the seeded fibrillation system presented here, faithfully recapitulates the phenomenon of species barriers. We therefore strongly support the hypothesis, that CWD cannot be transmitted to humans.

In summary, our *in vitro* seeding results are in complete agreement with epidemiological observations and *in vivo* studies.

In regard to the molecular mechanism of species barrier, we could show, that the seeding activity and thereby solely the molecular interaction of PrP^C^ and prion seeds is sufficient to explain the species barrier phenomenon in human prion diseases. Nevertheless, it cannot be excluded, that the transmissibility between species can be further influenced by cellular factors or processes within the living organism. As our seeds derive only from brain material of naturally occurring prion diseases, we have to point out, that our conclusions may not be transferable to any strain specific phenomena like tissue specificity or symptomless infections [7], [59].

With these restrictions in mind, our approach enables us to investigate or even predict uncertain or unknown species barriers by straightforward *in vitro* experiments compared to time consuming and expensive bioassays in transgenic rodents.
